# Activities on Facebook Reveal the Depressive State of Users

**DOI:** 10.2196/jmir.2718

**Published:** 2013-10-01

**Authors:** Sungkyu Park, Sang Won Lee, Jinah Kwak, Meeyoung Cha, Bumseok Jeong

**Affiliations:** ^1^Division of Web Science and TechnologyKorea Advanced Institute of Science and Technology (KAIST)DaejeonKorea, Republic Of; ^2^Graduate School of Medical Science and EngineeringKorea Advanced Institute of Science and Technology (KAIST)DaejeonKorea, Republic Of; ^3^Graduate School of Culture TechnologyKorea Advanced Institute of Science and Technology (KAIST)DaejeonKorea, Republic Of

**Keywords:** Facebook, Web application, depressive symptoms, online social network (OSN) activities, mental health, Internet

## Abstract

**Background:**

As online social media have become prominent, much effort has been spent on identifying users with depressive symptoms in order to aim at early diagnosis, treatment, and even prevention by using various online social media. In this paper, we focused on Facebook to discern any correlations between the platform’s features and users’ depressive symptoms. This work may be helpful in trying to reach and detect large numbers of depressed individuals more easily.

**Objective:**

Our goal was to develop a Web application and identify depressive symptom–related features from users of Facebook, a popular social networking platform.

**Methods:**

55 Facebook users (male=40, female=15, mean age 24.43, SD 3.90) were recruited through advertisement fliers distributed to students in a large university in Korea. Using EmotionDiary, the Facebook application we developed, we evaluated depressive symptoms using the Center for Epidemiological Studies-Depression (CES-D) scale. We also provided tips and facts about depression to participants and measured their responses using EmotionDiary. To identify the Facebook features related to depression, correlation analyses were performed between CES-D and participants’ responses to tips and facts or Facebook social features. Last, we interviewed depressed participants (CES-D≥25) to assess their depressive symptoms by a psychiatrist.

**Results:**

Facebook activities had predictive power in distinguishing depressed and nondepressed individuals. Participants’ response to tips and facts, which can be explained by the number of app tips viewed and app points, had a positive correlation (*P*=.04 for both cases), whereas the number of friends and location tags had a negative correlation with the CES-D scale (*P*=.08 and *P*=.045 respectively). Furthermore, in finding group differences in Facebook social activities, app tips viewed and app points resulted in significant differences (*P*=.01 and *P*=.03 respectively) between probably depressed and nondepressed individuals.

**Conclusions:**

Our results using EmotionDiary demonstrated that the more depressed one is, the more one will read tips and facts about depression. We also confirmed depressed individuals had significantly fewer interactions with others (eg, decreased number of friends and location tagging). Our app, EmotionDiary, can successfully evaluate depressive symptoms as well as provide useful tips and facts to users. These results open the door for examining Facebook activities to identify depressed individuals. We aim to conduct the experiment in multiple cultures as well.

## Introduction

Depression is one of the most common mental disorders. The lifetime prevalence of depressive disorder is 16.2% [1]; it commonly occurs in one’s early life and has a chronic course [2]. Depression is also related to reduced individual productivity and functional impairment that can cause a social burden [3]. It is predicted that depression will be the second leading cause of disease burden worldwide by 2020 [4]. The costs associated with depression and mental disorder have grown rapidly, and the National Institute of Mental Health reported in 2008 that major mental disorders cost at least $193 billion in the United States annually in lost earnings alone [5]. Therefore, depression has severe effects on individuals as well as on society.

Early diagnosis and prevention of depression can be an effective way to reduce depression-related problems because the length of the depressive episode is directly related to its recovery rate [6]. Therefore, significant effort has been spent on detecting symptoms of depression earlier in the general population. A number of campaigns have been proposed including National Depression Screening Day [7] and National Anxiety and Depression Awareness Week [8], which include offering free depression screening that can help find participants’ depressive symptoms in a prompt and easy way using several questionnaires. While these campaigns are an important step toward the early identification of potential patients, their main limitation lies in their potential bias; participants who have severe depressive symptoms, such as loss of energy or interest, might not attend the campaigns at all since to participate, they need to first go out. Therefore, by adopting our novel strategy, people can more easily access the new method, which actively adopts already widely spread online social media in contrast to conventional campaigns. This new approach may lessen the potential bias of conventional participation-oriented campaigns.

One possible screening method is using the large amount of data on online social networks (OSNs) [9]. OSN sites such as Facebook and Twitter, already used by hundreds of millions of users [10], have large-scale data that can be used to study health-related human behaviors in a cost-effective manner [11-13]. OSN data can be also used to reach and detect a large number of individuals with depression at low cost. Identifying the kinds of online social features that correlate with depression is crucial [9]. With the advent of OSN services, many attempts have been made to detect early symptoms of depression from online large-scale data [13-15].

Most research on finding depressive symptoms on OSNs have used words related to depression. Park et al [16] analyzed short text updates posted on Twitter to characterize the use of language related to depressive moods. The authors found that many online users openly disclose their depressive moods as well as treatment history in a public medium like Twitter. For instance, there was one tweet disclosing a detailed prescription as follows: “My doctor tries to give me birth control for depression, which works for me but I have so many side effects I would rather be moody.” Choudhury et al [17] examined linguistic and emotional correlates of postnatal changes in new mothers and built a statistical model to forecast significant postpartum behavioral changes using only prenatal observations. These studies show the potential application of social media in studying depressive symptoms, in particular, to understand the relationship between linguistic markers and mental disorder [18].

Moving beyond text analysis, many other approaches have been used to detect depressive symptoms online. Kotikalapudi et al [19] analyzed the patterns in the Web-browsing activities of college students that could signal depressive symptoms. Moreno et al [20] demonstrated that status updates on Facebook could reveal symptoms of major depressive episodes. Moreover, Rosenquist et al [21] found that levels of depression showed diffusion of up to three degrees of separation in a large social network, suggesting a wide influence of depressive symptoms through social links. It was also found that, in 2008, more than a quarter of Internet users searched for information about depression or mental health issues [22].

In this paper, we build on the abovementioned related work and make an effort to examine social network determinants of depressive symptoms. In doing so, we used data gathered from Facebook, which is currently the most widely used OSN in the world [23]. Even in Korea in 2011, Facebook surpassed the former most famous domestic OSN service, Cyworld, and became the most widely used social network [24]. Facebook contains a wide range of information about users, including demographic features such as age and gender, as well as social features such as friends list, like, interest, and location tagging. Together, these features could represent how a user maintains relationships online as well as offline [25,26]. Our research focus in this paper was to test whether a user’s mental health status can be predicted by the wide set of features available on Facebook.

For this study, we developed a mobile Web-based application for Facebook, called EmotionDiary, to recruit participants and seek markers of depressive symptoms on OSNs. The application offers two short self-report scales for measuring depressive symptoms: CES-D (Center for Epidemiologic Studies Depression) [27] and BDI (Beck’s Depression Inventory) [28], which are both well-proven approaches for measuring depressive symptomatology in the general population. This work aimed to identify depressive symptom-related features on Facebook that could distinguish depressed individuals from those who are not depressed. To determine whether the test overestimated or underestimated depressive symptoms and to gain a deeper understanding of user behaviors, this study was further assisted by face-to-face interviews of severely depressed individuals by a psychiatrist. Hence this paper provides both quantitative and qualitative findings toward detecting depressive symptoms in OSNs.

## Methods

### Overview

To demonstrate clearly the overall experiment and evaluation process, a detailed flowchart is provided in Multimedia Appendix 1.

### Application

**Figure 1:**
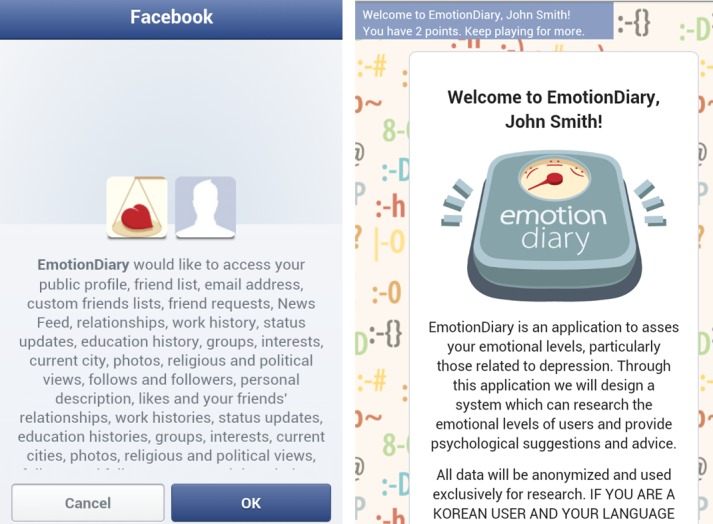
EmotionDiary screenshots: asking permission for data access and the welcome screen.

This study is based on a Facebook Web application that the authors developed and named EmotionDiary, which surveys depressive states of users and gathers demographic and social-activity data from Facebook. Upon accessing the app for the first time, users are shown a consent form that asks users for permission to access certain types of data from Facebook (see Figure 1). When users agree, the app becomes available and provides the depression questionnaires. The EmotionDiary app culled user data from Facebook every time a user accessed it. The app terminated for those users who did not consent.

**Figure 2:**
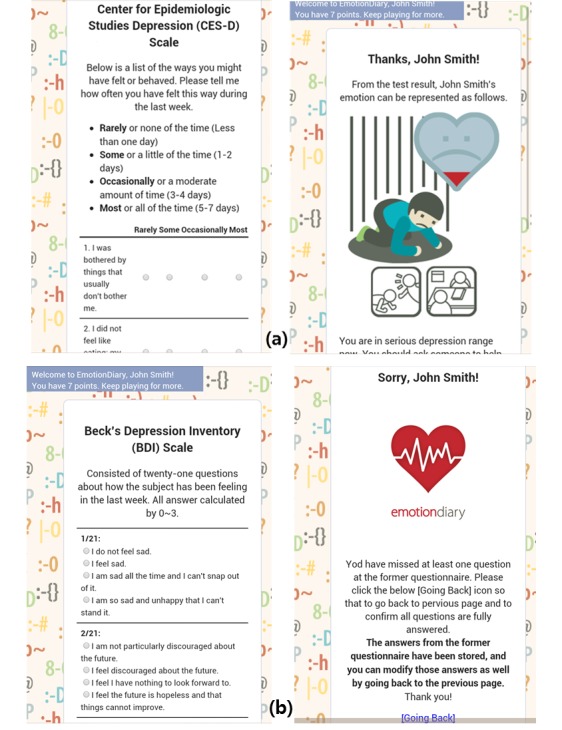
EmotionDiary screenshots: CES-D test and resulting feedback, and the BDI test and an error page when not all questions were answered.

EmotionDiary uses two authorized surveys, CES-D [27] and BDI [28], to screen depression. The CES-D self-report scale contains 20 simple questions, such that each questionnaire is rated 0 to 3 based on the frequency of depressive symptoms. The CES-D was developed to measure the symptoms of depression in community populations; it is commonly used in epidemiological studies. The Korean version of CES-D was standardized in 1993 [29]. The BDI is a self-report scale and consists of 21 questions about various domains of depressive symptoms including emotional, cognitive, physical, and motivational symptoms. Participants rate the severity of each item using a 0 to 3-point scale. The Korean version of the BDI scale used in this study was standardized in 1991, and its reliability and validity were verified [30]. We asked participants to take both surveys to ensure responses were consistent. Only those participants who completed both tests were included in our analysis. To avoid having any questions unanswered, when participants submitted the result with some missing answers, the app directed participants to go back and fill in all the remaining questions, as shown in the error page example in Figure 2.

The CES-D test was the first survey shown to participants, for which we provided three types of feedback (see Figure 2). Given that the score range of CES-D is between 0 and 60, we used the cutoffs of 16 and 25 suggested in previous studies [31,32]: a score of 16 to 24 represents probable depression, and 25 or higher represents definite depression or major depressive disorder. Each participant’s feedback included an infographic indicating the participant’s level of depression and possible recommendations to reduce their level of depression in everyday life. The precise score was not shown to participants. For instance, users whose CES-D scores fell below 16 received the following feedback: “Your score is in a normal range. However, you need to pay attention to your mental health since depression is a common disease. Taking a walk, exercising for 30 minutes, talking with friends, or sunbathing would be helpful to maintain a good mental state!”

**Figure 3:**
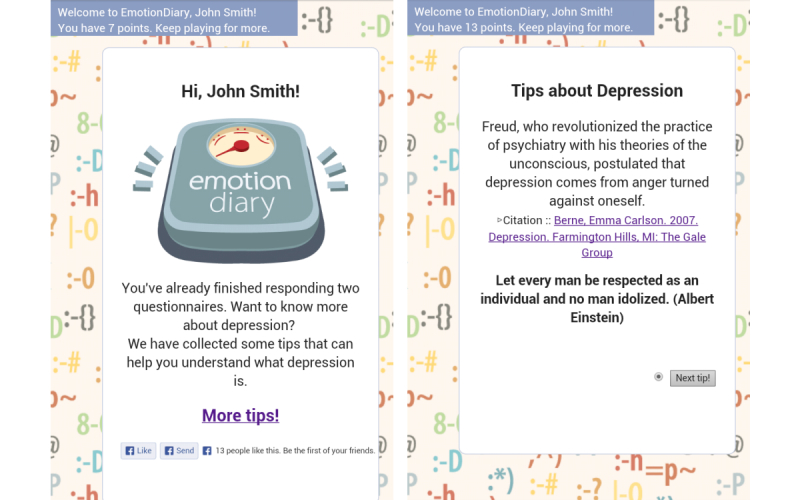
EmotionDiary screenshots: depression tips and facts.

Participants were taken to the BDI test after completing the CES-D test. We did not show any feedback for the BDI to avoid the same participant being judged to have a different depressive state from the CES-D. Once participants finished the BDI survey, they were led to view tips on improving their mental health. EmotionDiary contained 100 tips and facts pages: 40 tips to improve one’s mood and 60 general facts about depression (see Figure 3). The helping tips were selected from a self-help book on depression [33], and general facts were from a website entitled Random Facts [34]. Also, the app contained a points system, where participants could gain 1 point for accessing the app each time, 5 points for responding to each survey question, 3 points for viewing each tip, and so on. The point system was designed only to motivate users, and none of the participants were financially rewarded in any ways. Accumulating points and participating in viewing tips, which meant a participant was accessing the app and checking the individual tip and fact pages, can be associated with an acute depressive state rather than a trait. These two activities may better reveal the relationship between depression and OSN activities, because other Facebook social features contain cumulative data that cannot reflect acute behavioral changes.

To check stability of the app and compliance of the participants, before starting the actual research we conducted a pilot study with 28 participants different from those of the main experiment. As a result, we were assured that the app was able to handle data and that participants would be able to comply with the experiment (see Multimedia Appendix 2).

### Participants and Recruitment

**Figure 4:**
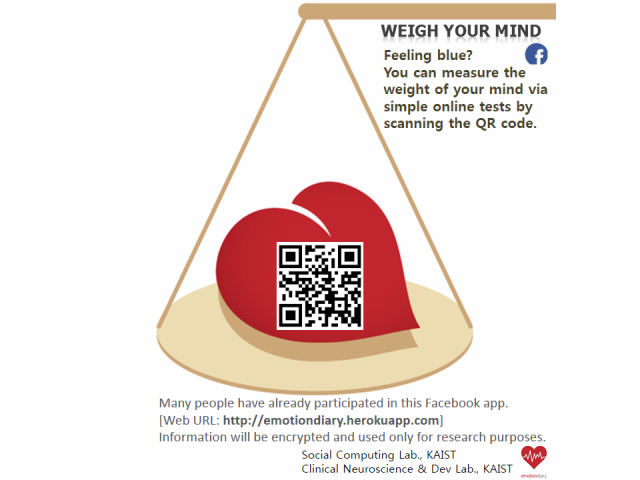
Flier for recruiting participants.

A total of 115 participants were recruited among undergraduate and graduate students at KAIST (Korea Advanced Institute of Science and Technology) who had Facebook accounts. Participants were randomly recruited through advertisement fliers posted on the major school buildings as well as through an online school BBS (Bulletin Board System) during a 2-week period from April 17-30, 2013 (see Figure 4). All participants joined and answered the questions on a voluntary basis without getting a financial reward. We fully explained the purpose of this study and the individual information that we would gather on Facebook. This study was approved by the KAIST Institutional Review Board (approval number: KH-2012-22).

### Data Description

The EmotionDiary app culled and stored three broad types of data from each user: (1) demographic information including name, age, and gender, (2) social-activity information such as friends list and likes from Facebook, and (3) app-generated data such as the depression score and the number of tips viewed. Table 1 summarizes the list of data gathered and used for analysis. In collecting data, Facebook’s API (Application Programming Interface) was used, similar to other studies on Facebook [35,36]. While the API can be used to gather a wide range of data, we primarily limited our focus to popular activity-related features, as in other studies. As described earlier, the three types of data were culled each time participants accessed the app from April 17-30, 2013.

### Statistical Analysis

First, Spearman rank correlation coefficient was used to test any relationship between the various features on Facebook and participants’ CES-D scores. Spearman correlation is a nonparametric method that can address outliers appropriately. After the correlation analysis, simple linear regression was applied to evaluate the detailed relationships between Facebook social features and CES-D scores.

Second, a Mann-Whitney *U* test was used to measure the differences in Facebook social features between participants in the probably depressed group and nondepressed group. This analytic method is useful when two groups do not follow normal distributions and when the number of participants is not large. In dividing users into two such groups (ie, normal and depressed), we jointly used the CES-D and BDI results and adopted the cutoff values proposed for screening from previous studies [37,38], with a CES-D score of 21 and the BDI score of 10. We chose the CES-D score of 21, rather than 16 or 25, because it has been known to be helpful in community-level screening [37]. Therefore, the depressed group consisted of individuals with a CES-D score≥21 and BDI≥10, and the normal group had a CES-D score<21 and BDI score<10.

### Face-to-Face Interview

Among participants who voluntarily participated in this study and had completed two screening scales, participants who garnered CES-D scores higher than 25 were invited for a 50-minute (approximately) interview with a psychiatrist at KAIST. This interview was conducted when participants accepted our invitation via a Facebook message. We chose a cutoff score of 25 because it can represent definite depression or major depressive disorder [31,32]. We sent a private invitation message to 7 of the 15 target interviewees through Facebook; two of them paid a visit to the clinic for the interview. The face-to-face interview had three purposes: (1) to evaluate participants’ depressive symptoms from a qualitative aspect and to find the relationship between detailed depressive symptoms and Facebook features, (2) to give appropriate feedback to participants with CES-D scores higher than 25, indicating the need for professional help, and (3) to measure the reliability and characteristics of online depression scales.

**Table 1:** Facebook social activity and app-generated features.

Feature		Feature description (ranges)
**User demographic**		
	Age	User’s age
	Gender	User’s gender information: male (0) and female (1)
**Facebook social activity**		
	Relationships	User’s relationship status: no data (excluded), single (coded as 1), in a relationship (2), engaged (3), married (4), widowed (0.5), separated (0.5), divorced (0.5)
	Groups	Number of groups to which a user belongs (including groups of which a user is an administrator)
	Group admins	Number of groups for which a user is an administrator
	Likes	Number of pages a user has marked as like
	Pending requests	Number of pending incoming friend requests
	Friends	Number of friends a user has
	Location tagging	Number of physical locations which a user has tagged
	Interests	Number of interest items listed on the user’s profile
	Activities	Number of activities listed on the user’s profile
	Events	Number of events a user is attending
**App-generated data**		
	Tips	Number of tip and fact sections list a user checked (total 100 tip and fact sections are offered; we counted the number of tip and fact sections user checked).
	Points	Accumulated points by participating in the app
	CES-D	Resulting score of the CES-D survey: range from 0 to 60
	BDI	Resulting score of the BDI survey: range from 0 to 63

## Results

### Overview

Of the 115 random participants who accessed the EmotionDiary app, 82 participants completed the CES-D survey (71.3%), and 56 of them also completed the BDI survey (48.7%). We focused only on the 56 participants who completed both questionnaires to select ones who truly participated and to exclude others who dropped off in the middle of the experiment. Furthermore, we set an acceptable range on the number of friends, 0-1000, and groups in which they participated, 0-50, excluding those users who exceeded these limits because such users may be considered “microcelebrities” who exhibit different behavioral patterns from ordinary users on Facebook. Through this screening process, one male participant was excluded from analysis due to having more than 1000 Facebook friends. The remainder of this paper describes the analysis of the 55 participants.

Of the 55 participants retained for further analyses, 40 were males aged 19-36 (mean age 24.89, SD 4.35) and 15 were females aged between 19-28 (mean age 23.33, SD 2.17). Table 2 summarizes the mean and quartile values of participants based on their demographic, social activity, and app features. The table also shows the first quartile (25th) and the third quartile (75th) values to indicate the overall distribution of each feature. Most participants had no “activities” and “events”; hence, we removed these features from the analysis.

**Table 2:** Mean values of participants’ Facebook social activity and app features (N=55).

Feature			Mean (SD)	1^st^ quartile	3^rd^ quartile
**User demographic**					
		Age	24.43 (3.90)	22	26
		Gender	M=40, F=15		
**Facebook social activity**					
		Relationships (n=29)	0.75 (0.79)	1	2
		Groups	13.25 (9.02)	7	19
		Group admins	0.73 (1.22)	0	1
		Likes	46.11 (60.61)	11	54
		Pending requests	6.24 (8.62)	0	8
		Friends	315.62 (182.80)	179	426
		Location tagging	12.8 (10.03)	3	25
		Interests	1.13 (2.90)	0	1
**App-generated data**					
	Tips		2.93 (4.02)	0	4
	Points		19.96 (12.04)	11	23
	CES-D		16.84 (9.56)	10	25
	BDI		11.03 (9.29)	3	16

### Relationship Between Facebook Social Features and CES-D Scale

To understand the relationship between the various social-network features and one’s depressive state, we examined Spearman rank correlation. Rather than comparing absolute values, we used the rank scale to compensate for the broad range of variations among different features. Table 3 shows the resulting correlation for a representative 9 features. Not all features showed correlation with the CES-D score, such as age (omitted in the table), yet certain features had meaningful correlations. The number of location tags had a negative correlation with the CES-D score (*P*=.045), whereas the accumulated app points and the number of viewed tips had positive correlations (*P*=.04 for both cases). Additionally, the number of friends showed a marginally significant negative correlation (*P*=.08). Some of the features that showed weak trends revealed a stronger tendency when we examined the Spearman correlation among the same features with BDI instead of CES-D scores. For instance, the relationship between BDI scores and the number of friends, app points, and app tips features turned out to be significant (see Multimedia Appendix 3).

Next, we performed a simple linear regression between the BDI and CES-D scores to determine whether participants responded to questionnaires consistently. The BDI and CES-D scores are known to have highly positive correlation [39]. Our linear regression result also confirms the significant relationship between CES-D and BDI (see Multimedia Appendix 4); the Spearman correlation coefficient was 0.839 with *P*<.001, which indicates that participants were consistent in responding to the two surveys. However, we also saw a few outliers whose CES-D and BDI scores were outside the confidence interval in linearity. Hence, in the following group comparison, we excluded these outliers to further increase the level of credibility in data and examine intrinsic traits of individuals in the probably depressed group that can be discriminated from those in the nondepressed group.

**Table 3:** Spearman rank correlation coefficients between Facebook social features and the CES-D scale.

	Groups	Group admins	Interests	Likes	Pending requests	Location tagging	Friends	App points	App tips
Spearman’s rho	-0.109	-0.104	-0.210	-0.220	-0.074	-0.272	-0.237	0.274	0.278
*P* value	.43	.45	.12	.11	.59	.045	.08	.04	.04

### Difference in Facebook Social Features Between Depressed and Nondepressed Groups

To find differences between depressed and nondepressed individuals, we examined per-group traits by joining the CES-D and BDI scores, as described in the statistical analysis. In doing so, an additional 13 participants were excluded since their CES-D and BDI scores did not match. For instance, some users had a CES-D score greater than or equal to 21 but a BDI score lower than 10. Due to this additional filtering, a total of 42 participants were chosen to be participants for group comparison, where 16 were classified as the probable depression group and 26 as the nondepressed group.

Table 4 shows the results of the Mann-Whitney *U* test between the probably depressed and nondepressed groups. We found that certain Facebook activities had predictive power in distinguishing depressed and nondepressed groups. Among the features, the number of location tags showed marked differences (*P*=.07) in that users in the nondepressed group were far more likely to have a higher number of location tags. In addition, the total accumulated points in EmotionDiary (*P*=.03) and the number of tips viewed (*P*=.01) were also significantly different between the two groups: the depressed group was far more likely to engage in these activities than its nondepressed counterpart.

**Table 4:** Results of the Mann-Whitney *U* test of Facebook social features between those who were probably depressed^a^ (n=16) and nondepressed^b^ (n=26).

	Probably depressed^a^ mean (SD)	Nondepressed^b^ mean (SD)	Z score(two-tailed)	*P* value
Groups	12.56 (8.90)	13.46 (9.35)	-0.207	.84
Group admins	0.75 (1.18)	0.65 (1.26)	-0.365	.72
Likes	34.06 (35.65)	56.46 (79.73)	-0.505	.61
Pending requests	3.50 (6.55)	6.54 (8.67)	-1.122	.26
Friends	253.87 (178.75)	338.42 (183.30)	-1.450	.15
Location tagging	9.62 (9.81)	14.53 (9.78)	-1.790	.07
Interests	0.38 (1.09)	1.62 (3.91)	-1.475	.14
App points	24.69 (14.98)	15.26 (6.43)	-2.229	.03
App tips	4.50 (4.99)	1.38 (2.14)	-2.449	.01

^a^Probably depressed: CES-D≥21 and BDI≥10.

^b^Nondepressed: CES-D≤20 and BDI≤9.

### Face-to-Face Interview

One psychiatrist evaluated the depressive symptoms of 2 participants through the Hamilton Depression Rating Scale (HAM-D) [40] within 2 weeks of the EmotionDiary test. All participants showed symptoms of depression including depressed mood, feelings of guilt, insomnia, and anxiety. Their HAM-D scores were higher than 7 points, which is a reliable cutoff point for depression [41]. The participants showed moderate depressive symptoms on both the HAM-D and CES-D; they were judged to be in a chronic depressive state (see Table 5). One participant (Participant A) mainly complained about depressed mood, while the other participant (Participant B) reported a severe loss of interest and exhibited decreased activity in Facebook. This participant used the likes and location-tagging features only once and twice, respectively, and belonged to the 1^st^ quartile in the amount of activities among all Facebook participants. In general, Facebook activities of the 2 participants in location tagging and number of friends, related to depression (shown in Table 3), were relatively minimal. Most values of these features were below the median value.

**Table 5:** Characteristics of participants.

	Sex	CES-D	HAM-D	Likes	Friends	Location tagging	App tips	Remarks
Participant A	M	32	17	46	19^a^	15	1^b^	Chronic depressive state
Participant B	M	25	17	1^a^	138^a^	2^a^	0^a^	Chronic depressive state

^a^Below 1^st^ quartile value.

^b^Below median value of 55 participants.

## Discussion

### Principal Findings

OSNs like Facebook have become a primary platform of communication in today’s societies. In particular, among young adults, most college students are known to have accounts on OSNs [42] and conduct substantial amounts of interpersonal activities through OSNs [43]. Given their widespread use, this paper explored the idea of using OSNs for a cost-effective and large-scale screening of depression, under the assumption that depressed individuals would exhibit distinguishing behavioral markers online compared to their nondepressed counterparts. Toward this goal, we developed a Facebook Web application called EmotionDiary, which provided users with surveys for depressive symptoms and tips on depression. Based on the analysis driven by data from 55 participants, although preliminary, we found that several Facebook features are associated with depressive symptoms.

First, the number of viewed pages of tips and facts in EmotionDiary is positively correlated with the severity of depression, in that depressed participants read more tips than their nondepressed counterparts. These results suggest that activities in the EmotionDiary app can be more useful and consistent as an acute state marker for depression than other Facebook social features. This may be because many OSN features cannot reflect the acute state of one’s mental health since they are formed over a long period of time. Using an app, in contrast, can better reveal the dynamic state of the users. In previous studies, people with psychiatric problems were found to use the Internet to get mental health-related information more frequently than an average person [44,45]. Reading more tips may be related to efforts and interests to overcome their depression. From this finding the app may have the potential to enable successful interaction with people who have depressive symptoms and need some help. One notable observation we made was the difference in behaviors related to chronic quality of depression. From the interviews, although numbers of interviewees were limited, we found that participants with chronic depression (Participants A and B, shown in Table 5) checked tips less frequently than average. Thus, the significant increase in the number of viewed tips for the depression group (see Table 4) indicates that most depressed individuals in our study were experiencing acute stressful events rather than being chronically depressed.

Second, participants who had many Facebook friends showed a low likelihood for depression. Participants with depressive symptoms who suffered from recurrent or chronic depression might not want to increase their number of OSN friends. In previous studies, severely depressed participants did not try to enhance their social network [46]. Also, our observations may be related to findings from other research that shows that Facebook friends can play a role and give social support, which is a key protective factor for depression [47,48]. Previous studies also showed that interpersonal relationships in Facebook are helpful in improving depression [49]. Having many friends on Facebook may allow users to be involved in more communication with other people, thereby reducing depression.

Third, the number of location tags is negatively correlated with one’s severity of depression, in that nondepressed individuals were more likely to have location tags. Location tagging is a function commonly used when people visit a new interesting place, for instance, a nice restaurant, park, or concert. Location tagging is the function that requires users to enter their real-world experiences, as one needs to visit a specific place that is notable enough to be worth sharing on Facebook. In fact, loss of interest or pleasure is known to be a key symptom of depression in young adults [50], and this may explain why depressed individuals appear to be less exposed to new experiences (ie, their activities or interests are decreased due to depression) or less likely to “share” their experiences with others. Thus, the decrease in number of location tags might reflect these anhedonia-related symptoms of depression. Additionally, peculiar characteristics related to social withdrawal, such as “Hikikomori”, a Japanese term meaning young adults who withdraw from social life and seek extreme degrees of isolation [51], can affect Facebook features too. Recent findings may show this explanation is possible [52]. The relationships between personal characteristics and Facebook social features should be clarified in future studies. It is possible that the number of location tags can be simply related to total duration of Facebook use, in that the longer the duration of Facebook use, the greater the location tagging. Nonetheless, our findings suggest the possibility that location tagging can be used to mark the status of depressive symptoms.

Interestingly, depressed users show a decreasing tendency to use the like feature, although not to a significant degree, as was seen in the decrease of location tagging. The like feature can show positive empathy, interest, or agreement about someone else’s status update. Although the like feature can be related to a decrease in interests in general, this feature is much easier to use than location tagging because location tagging requires physical activity. This difference may explain the weaker correlation between the like feature and depression.

Finally, the qualitative interviews suggested a possibility that differences in individual depression symptoms can affect behaviors on Facebook. For example, participants reporting severe loss of interest showed low activity in likes and location tagging, while participants with chronic depressive symptoms read very few tips. These results may reflect specific types of depressive symptoms or subtypes of depression that can influence which OSNs features users engage with. In fact, depression is a heterogeneous disease and evaluating the subtypes of depression is an important challenge [53,54]. For example, major depressive disorder has several subtypes including melancholic and atypical types, according to the Diagnostic and Statistical Manual of Mental Disorders (DSM-IV). Different subtypes have differences in symptom domains, as well as epidemiological backgrounds [55], clinical courses [56], endocrine profiles [57], and treatment responses [58]. Therefore, if features of OSNs can give additional information about the specific types of depression more precisely, that can also provide clues toward resolving several debated issues in depression research. We can also consider assessing the answers for each survey question for future studies, since each question is designed to reflect a specific topic such as diet or insomnia, thereby clarifying participants’ depressive attributes in more detail.

Results from our interviews could not be generalized to the whole population facing depressive symptoms and could not be evaluated to provide reliability of online screening tests since the number of interviewees was limited (n=2). However, low response rates might reflect the problem of low accessibility to existing face-to-face evaluation. In practice, we were able to contact only 7 of 15 participants who scored greater or equal to 25 in CES-D scores, since the 8 did not want to receive Facebook messages; only 2 of 7 participants who successfully received messages agreed to visit for an interview. Thus, novel approaches, such as EmotionDiary, would be helpful to evaluate and manage depressive symptoms of people who do not want to unveil their depressive symptoms in person.

### Limitations and Future Plans

There are several limitations to our study. First, the Facebook social activities we analyzed were accumulated before the CES-D test; we could not measure the patterns of changes in Facebook social features. Therefore, additional prospective studies are needed to mitigate this limitation. Second, the study was conducted with a particular demographic of students in KAIST, which is top-ranked university in South Korea. Also, KAIST is attended mainly by young male students. This limits us from generalizing the findings to the general population and limits the predictive power of Facebook features. Additionally, we think cultural differences could affect our result, as our findings may include Korea-specific trends in the way people use Facebook. However, we could not find ample studies or evidence on how cultural differences affect one’s behavior online related to depressive symptoms. To generalize our results, further studies that are focused on transcultural similarities and differences are needed to evaluate depressive mood of groups from various ethnicities and ages. This study is also limited by the relatively small number of participants (N=55) who completed both CES-D and BDI tests. We think much additional research from various perspectives will be necessary to evaluate symptoms and moods appropriately, using online social features.

The number of participants we interviewed is small (n=2), in particular because our aim was to gain quick insights into how participants with depressive symptoms perceived our application. Although the results of interviews could not explain general patterns of groups with depressive symptoms, we could confirm the low accessibility of face-to-face evaluation. We would like to recruit more interviewees or to use other qualitative measurements in future studies. While we did not employ any explicit rewards in EmotionDiary, an appropriate incentive mechanism may be added to facilitate participant recruitment and the successful completion of depression surveys.

Despite the abovementioned limitations, to the best of our knowledge, our study is the first attempt to identify an association between social features on Facebook and users’ depressive symptoms. By analyzing Facebook-related depressive traits, we tried to understand human behaviors in a social relationship that could predict depressive moods. Therefore, this study is an important step toward the problem of large-scale screening of depression on OSN platforms. As OSNs are becoming a primary communication platform for more people, we believe mobile and Web-based applications like EmotionDiary can serve an important role in increasing the awareness of depressive symptoms in our society and promoting positive health behaviors in an unintrusive manner.

## Multimedia Appendix 1

Flowchart of the overall experiment and evaluation process.

## Multimedia Appendix 2

Pilot study concepts and interviewee characteristics.

## Multimedia Appendix 3

Spearman rank correlation coefficients between the online social features and the BDI scores.

## Multimedia Appendix 4

Relationship between the CES-D and BDI scores of 55 participants.

## Abbreviations

API: application programming interface
App: application
BBS: bulletin board system
BDI: Beck’s Depression Inventory
CES-D: Center for Epidemiologic Studies-Depression
DSM-IV: Diagnostic and Statistical Manual of Mental Disorders
HAM-D: Hamilton Depression Rating Scale
KAIST: Korea Advanced Institute of Science and Technology
OSN: online social network
