# Supplementary figures and images for: Activities on Facebook Reveal the Depressive State of Users
Source: J Med Internet Res. 2013 Oct 1;15(10):e217. doi: 10.2196/jmir.2718 (PMC3806432; doi:10.2196/jmir.2718)

Multimedia Appendix 1. Flow chart of the overall experiment and evaluation process

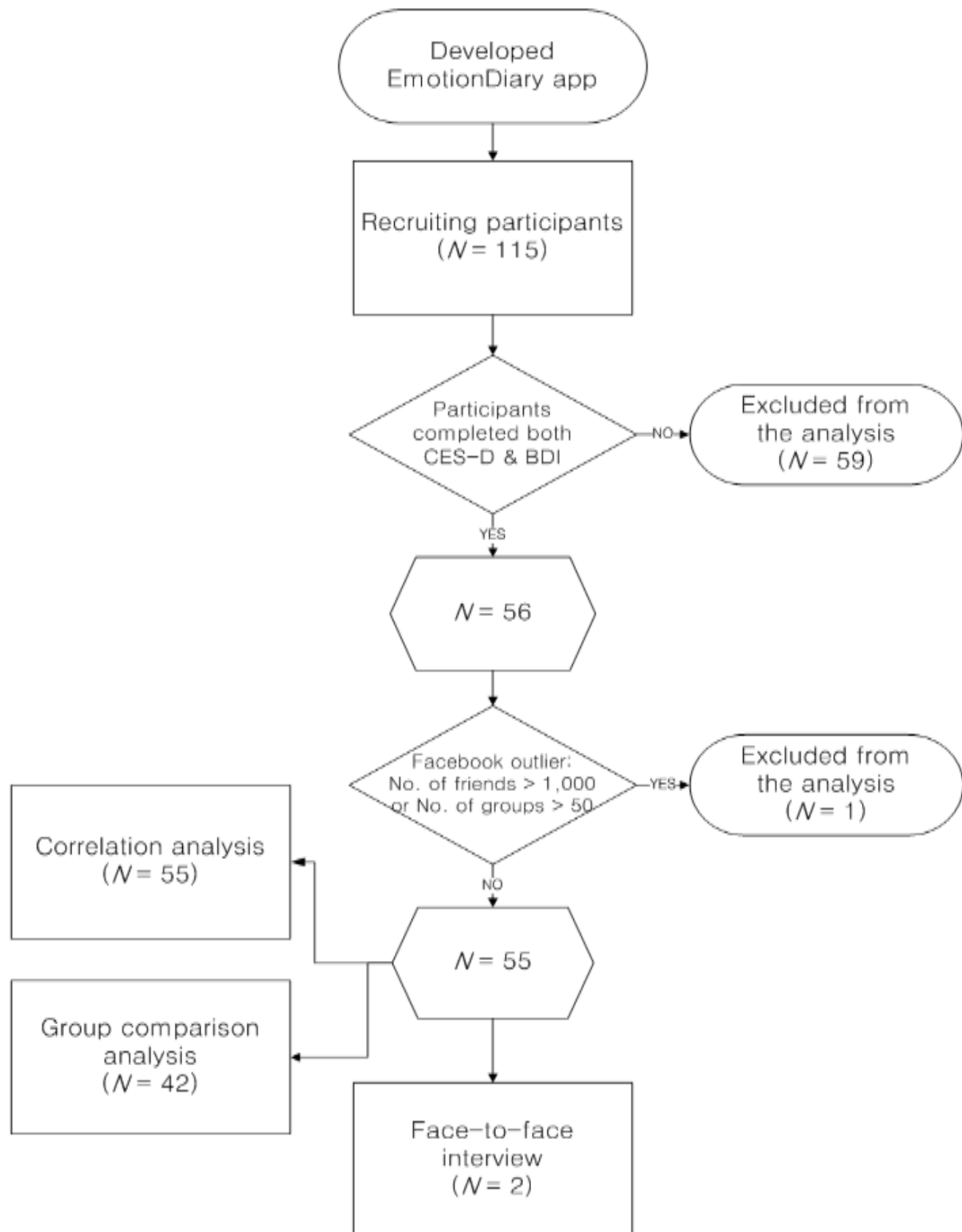

Supplement: Supplementary file 1 [file jmir_v15i10e217_app1.pdf]

**Multimedia Appendix 4.** Relationship between the CES-D and BDI scores of 55 participants

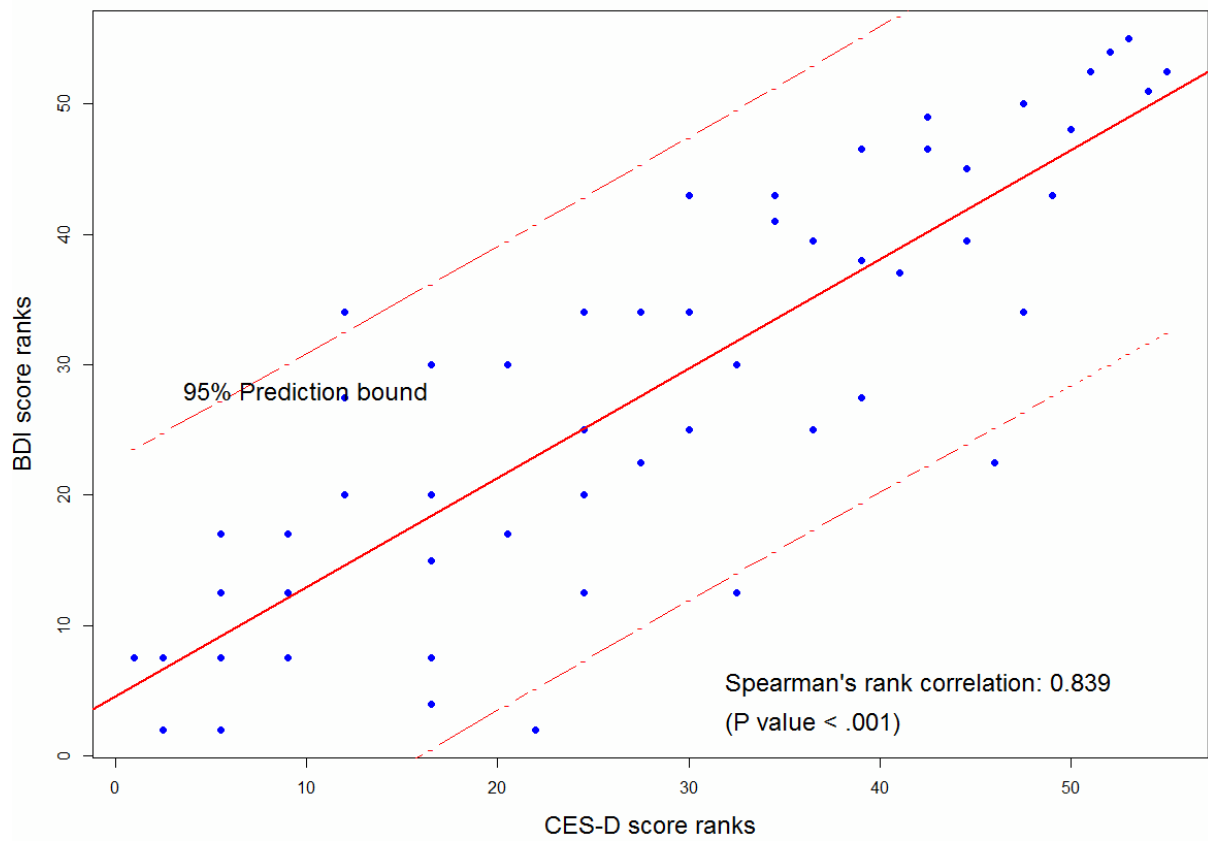

Supplement: Supplementary file 4 [file jmir_v15i10e217_app4.pdf]
