# Supplementary material for: Activities on Facebook Reveal the Depressive State of Users
Source: J Med Internet Res. 2013 Oct 1;15(10):e217. doi: 10.2196/jmir.2718 (PMC3806432; doi:10.2196/jmir.2718)
Supplement: Supplementary file 2 [file jmir_v15i10e217_app2.pdf]

Multimedia Appendix 2. Concepts of the pilot study and the characteristics of interviewees

Objectives:

Our main goal of pilot study was to evaluate the stability of EmotionDiary system and compliance of participant. In addition, we tried to find the social features related to depression.

Methods:

During from Feb 2 to Feb 15, we recruited 28 participants on Facebook, 22 males (mean age=28.7± 2.2) and 6 females (mean age=26± 1.3), We measured their depressive mood by Center for Epidemiological Studies-Depression (CES-D) scale, and a trained psychiatrist interviewed individuals with high CES-D scales to verify their depressive states using Hamilton Depression Rating Scale (HAM-D).

Results and Conclusion:

All 28 participants finished the study; only 5 participants showed depression. Some Facebook social features, such as likes, related to positive interactions were reduced in depressed group. Also, one psychiatrist had an interview with two subjects, and online CES-D score was relatively reliable when compared to HAM-D score. Compliance and stability of EmotionDiary were good, and further study for more subjects should be applied to measure the depression related features.

Table. Characteristics of face-to-face interviewees in pilot study

	Sex	CES-D	HAM-D	Likes	Friends	Location tagging	App-tips	Remarks
Participant C	M	26	13	22	357	13	10	Severe stress when checked CES-D (acute)
Participant D	F	25	9	11	35	10	10	Severe stress when checked CES-D (acute)
