# Supplementary material for: Activities on Facebook Reveal the Depressive State of Users
Source: J Med Internet Res. 2013 Oct 1;15(10):e217. doi: 10.2196/jmir.2718 (PMC3806432; doi:10.2196/jmir.2718)
Supplement: Supplementary file 3 [file jmir_v15i10e217_app3.pdf]

**Multimedia Appendix 3.** Spearman's rank correlation coefficients between the online social features and the BDI scores

| | Facebook Social Features | | | | | | | | |
|---|---|---|---|---|---|---|---|---|---|
| | **Groups** | **Group admins** | **Interests** | **Likes** | **Pending requests** | **Location tagging** | **Friends** | **App- points** | **App- tips** |
| **Spearman's rho** | -0.127 | 0.011 | -0.160 | -0.136 | -0.043 | -0.266 | -0.288 | 0.374 | 0.385 |
| ***P* value** | .36 | .94 | .24 | .32 | .76 | .05 | .03 | .005 | .004 |

The number of location tagging and friends was negatively correlated with BDI score ($P$ = .05 and $P$ = .03 respectively), while the App-points and the number of App-tips viewed had positive correlation ($P$ = .005 and $P$ = .004 respectively).
